# Role of QuantiFERON-TB Gold, Interferon Gamma Inducible Protein-10 and Tuberculin Skin Test in Active Tuberculosis Diagnosis

**DOI:** 10.1371/journal.pone.0009051

**Published:** 2010-02-04

**Authors:** Basirudeen Syed Ahamed Kabeer, Balambal Raman, Aleyamma Thomas, Venkatesan Perumal, Alamelu Raja

**Affiliations:** 1 Department of Immunology, Tuberculosis Research Centre (ICMR), Chennai, Tamil Nadu, India; 2 Department of Clinic, Tuberculosis Research Centre (ICMR), Chennai, Tamil Nadu, India; 3 Department of Statistics, Tuberculosis Research Centre (ICMR), Chennai, Tamil Nadu, India; University of Melbourne, Australia

## Abstract

**Background:**

The measurement of Interferon gamma or Interferon gamma inducible protein (IP)-10 in antigen stimulated blood samples is suggested as an alternative method for latent tuberculosis (TB) diagnosis. Nonetheless, their role in active TB diagnosis, particularly in TB endemic settings is yet to be defined. In this study, the sensitivities and specificities of Interferon gamma release assay (IGRA), IP-10 assay and tuberculin skin test (TST) in detecting active TB cases were assessed in human immunodeficiency virus (HIV) sero-negative TB patients and healthy controls respectively.

**Methods/Principal Findings:**

A total of 177 adult TB patients and 100 healthy controls were included for this study. QuantiFERON-TB Gold In-tube (QFT-IT) method was used to analyze the sensitivity and specificity of IGRA. QFT-IT, IP-10 and TST yielded the diagnostic sensitivities of 90.6% (95%CI: 86.3%–94.9%), 92.5% (95%CI: 88.6%–96.4%) and 68.9% (95%CI: 60.6%–77.2%) and specificities of 55% (95% CI: 35.2%–54.8%), 48% (95% CI: 38.2%–57.8%) and 75.5% (95% CI: 66.8%–84.2%), respectively. The extent of pulmonary involvement or presence of diabetes mellitus did not appear to influence the sensitivities of any of these tests. The combination of any of the two tests among QFT-IT, IP-10 and TST showed >98% sensitivity among smear negative cases and particularly the combination of IP-10, TST and smear microscopy showed 100% sensitivity, however, the specificity was decreased to 44.8%.

**Conclusions/Significance:**

QFT-IT and IP-10 were highly sensitive in detecting active TB cases. The combination with TST improved the sensitivity of QFT-IT and IP-10 significantly. Although the higher sensitivity of combination of QFT-IT/IP-10 and TST may be useful in active TB diagnosis, they are limited by their poor specificity due to the high prevalence of latent TB in our settings.

## Introduction

Tuberculosis (TB) remains the single infectious disease, causing the highest mortality in humans, leading to 3 million deaths annually, about five deaths every minute [1]. The alarming increase in morbidity and mortality due to TB indicates the need to strengthen control measures. Control of this disease depends largely on early detection and treatment of active cases. Smear microscopy and nucleic acid amplification test are the two currently available rapid confirmatory tests for active TB disease diagnosis, but both are limited by their poor sensitivity [2], [3]. Another confirmatory test for active TB disease is culturing the mycobacterium, but it requires several weeks to obtain the results [2].

A recent break through in TB diagnosis is the introduction of interferon gamma release assay (IGRA) in which the production of interferon gamma (IFN-γ) in response to *Mycobacterium tuberculosis* specific antigens is measured. Three commercial kits based on the IGRA principle are available: T-SPOT.TB, QuantiFERON-TB Gold, QuantiFERON-TB Gold in-tube (QFT-IT). T-SPOT.TB and QuantiFERON TB Gold assays use only Early Secretory Antigenic Target (ESAT)-6 and Culture Filtrate Protein (CFP)-10, whereas an additional antigen TB 7.7 is incorporated in QuantiFERON kits. The currently available data suggest that IGRAs are less influenced by prior BCG vaccination and environmental mycobacteria [4]. Though IGRAs are offered for latent TB diagnosis (LTBI), studies are ongoing to define their role in diagnosis of active TB disease too.

The sensitivity of IGRAs is reported to be higher in detecting active TB patients in low TB endemic countries [5]–[11]. A recent study conducted in urban hospitals in United Kingdom using ELISPOT, reported that IGRA in combination with TST can be used to rule out the suspicion of active TB disease among clinically suspected subjects [12]. However, the data from high TB endemic countries are limited. Thus, further studies are needed to validate the role of IGRA in the diagnosis of TB in high endemic settings.

The data available from high TB endemic countries clearly evidence that sensitivity of IGRA is relatively lower in low TB endemic countries [13]–[16]. In this situation, various attempts have been made to enhance the sensitivity of IGRA and one of them is evaluating additional or alternative biomarkers.

Earlier studies conducted to identify the new diagnostic markers suggest that IFN-γ inducible protein (IP)-10 is a potential biomarker for TB diagnosis [11], [17]–[20]. IP-10 is a pro-inflammatory chemokine, expressed by monocytes and macrophages [21]. Upon contact with viral or bacterial agents, antigens or mitogens, the pro-inflammatory cytokines like IFN-γ stimulates expression of IP-10 [22]–[24]. However, none of the studies have been conducted in high endemic settings to compare the sensitivity and specificity of IGRA and IP-10 assays for active TB diagnosis.

In this study, we aimed to assess the sensitivities and specificities of QFT-IT, IP-10 and tuberculin skin test (TST) among human immunodeficiency virus (HIV) negative individuals to define their role in active TB diagnosis in our settings, where TB is endemic.

## Materials and Methods

### Study Participants

This study was approved by the Scientific Advisory Committee and Institutional Ethical Committee of Tuberculosis Research Centre, Chennai. The recruitment of study subjects was done between November 2007 and October 2008. All the study subjects were informed about the study procedure and the volunteers were assessed for this study. Individuals with previous history of TB, those who underwent TST in the past 16 months, those with HIV infection, silicosis, end stage renal disease, leukemia/lymphoma or those under immunosuppressive therapy were excluded from the study. Eligible subjects consenting to the study were recruited into one of the following two groups.

#### (i) Healthy controls

This group consisted of healthy adult subjects age ranged ≥18 years, apparently free of TB symptoms and did not have close family contact of TB. All these subjects were recruited from offices, colleges located at Chennai and near by villages to Chennai. Since our setting is endemic to TB [25], to rule out the suspicion of active TB disease, all the subjects were asked to give three sputum samples and subjected to radiological examination.

#### (ii) Pulmonary TB (PTB) patients

This group consisted of pulmonary TB patients who were recruited from Revised National Tuberculosis Control Program (RNTCP) centers. The subjects attending the RNTCP centre with the abnormalities, suggestive of PTB in the chest X-ray and/or positive results for sputum microscopy were assessed. Three sputum samples were collected from all these study subjects and all were subjected to radiological examination. The collected sputum samples were processed [26], stained for acid fast bacilli (AFB) microscopy by Ziehl-Neelsen method and cultured in Lowenstein Jensen (Biomerieux Inc., Marcy I'Etoile, France) and in liquid MP BacT medium (Biomerieux Inc., Marcy I'Etoile, France). The presence of M. tuberculosis in the positive culture samples was further confirmed by Gen-probe based PCR (Biomerieux Inc., Marcy I'Etoile, France) method. The presence of active TB was defined as positive for sputum smear microscopy and/or positive for M. tuberculosis in sputum culture and/or abnormality suggestive of TB in chest x-ray. The patients with smear and culture negative results but had abnormalities on chest x-ray, which was suggestive as TB, were given broad spectrum antibiotics for three weeks. The non-responders were then treated with anti-tuberculous therapy (ATT) and followed up for at least two months. If the subject responded to ATT, then he/she was considered as PTB patients as per RNTCP guidelines [27].

Blood was drawn from all the recruited study subjects for total blood count, HIV testing, random blood sugar levels, QFT-IT and IP-10 assay. Then, the TST was carried out.

### HIV Testing

The HIV status was confirmed by 2 rapid tests (Retroquic Comb Aids-RS, Span Diagnostics, India and HIV TRI-DOT, J. Mitra & Co, India). When a serum was positive for both tests, it was considered as HIV positive. If a serum was positive for only one EIA (which was rare), Western Blot was done as confirmatory test.

### Blood Sugar Levels

Random plasma glucose levels were measured by GOD-POD method using Olympus AU 400 auto analyzer (Olympus GmbH, Hamburg, Germany) with Olympus reagents. The normal range for random blood sugar was considered as 80–140 mg/dl as per kit instructions [28].

### QFT-IT

The IFN-γ release assay was performed using QFT-IT In-tube test (Cellestis Ltd., Victoria, Australia). One ml of blood was taken in each of the three tubes precoated with TB–antigen, phytohemaglutinin (PHA) for the positive control or no antigen for the negative control. The blood samples were drawn between 10 and 11 AM and taken to the lab within 2 hours of phlebotomy. The tubes were incubated for 16–24 hours at 37°C and plasma were collected after centrifugation and stored at 4°C until assayed. Within 2 weeks of time, QFT-IT enzyme linked immunosorbant assay (ELISA) was carried out. The test results were interpreted using software supplied by the manufacturer (Cellestis Ltd., Victoria, Australia) and the cut-off point for the diagnosis was followed as per manufacturer's instruction. If the IFN-γ secretion in response to TB antigen, after subtracting nil control IFN-γ, was ≥0.35 IU/ml, it was considered as positive for QFT-IT and if the value was <0.35 IU/ml, it was considered as negative. If the negativity was associated with poor PHA response (i.e. IFN-γ secretion in response to PHA was <0.5 IU/ml), it was considered as indeterminate or invalid result for QFT-IT. The subjects with IFN-γ secretion >8.0 IU/ml in the nil control samples were also considered as indeterminate for QFT-IT.

### IP-10 Assay

The IP-10 levels were measured in duplicates in the supernatants collected from QFT-IT tubes using BD opt EIA kits (BD Biosciences, USA) as per manufacturer's instructions [29]. Briefly, 100 µl of capture antibody (mouse anti human IP-10 monoclonal antibody) at the recommended concentration was coated in the 96-well flat bottom polystyrene plates (NUNC maxisorp, Roskilde, Denmark). After overnight incubation, the excess antibodies were washed off using PBST. The sample was added to the plate, incubated for 2 hours and then the plates were washed off. The secondary antibody (biotinylated anti human IP-10 monoclonal antibody) conjugated with HRP was incubated for 1 hour and then the excess antibodies were washed off. Then tetra methyl benzidine (TMB) was used as substrate and incubated for 30 minutes and the reaction was arrested by the addition of 2 N H_2_SO_4_.

### Tuberculin Skin Test

The 2 TU (tuberculin unit) of purified protein derivative (PPD) RT23 (Statens Serum Institute, Copenhagen, Denmark) was injected intradermally by Mantoux method and the induration was measured between 48–72 hrs after PPD injection by trained professionals. The cut-off point for TST positivity was considered as 10 mm for this study.

### Statistical Analysis

Data were analyzed using GraphPad Prism version 5.00 for Windows (GraphPad Software, San Diego, California, USA) and GraphPad software available in their website (www.graphpad.com/quickcals.cfm). Kruskal-Wallis test was carried out to calculate the differences of IFN-γ and IP-10 levels between the groups. The proportion of positivity between TST and QFT-IT or IP-10 was compared using Fisher exact test. The comparison between the QFT-IT and IP-10 was done by using McNemar Chi-square tests, by treating the data as paired.

## Results

A total of 277 study subjects were recruited during the study period. There were 100 healthy controls and 177 PTB patients. Blood was unavailable for 2 PTB patients. Tuberculin skin test results were unavailable for 6 healthy controls and 58 (32.8%) PTB patients.

All the subjects were negative for HIV infection and demographic profile of those subjects is given in **Table 1**. All the healthy controls were negative for sputum smear microscopy and sputum culture. In PTB, sputum smear microscopy was positive in 151 (85.3%) subjects and sputum culture was positive in 162 (91.5%) subjects. All the sputum smear positive cases were positive for sputum culture.

**Table 1:** Demographic and baseline parameters of study subjects.

Category	HA	PTB
Number of subjects	100	177
Sex, N (%)		
Male	60 (60)	134 (75.7)
Female	40 (40)	43 (24.3)
Age, Median in years (Range; IQR)	29 (18–85; 22,50)	37 (18–70; 27,47)
Smear, N (%)		
Positive	0 (0)	151 (85.3)
Negative	100 (100)	26 (14.7)
Culture, N (%)		
Positive	0 (0)	162 (91.5)
Negative	100 (100)	15 (8.5)
Diabetes mellitus, N (%)		
Present	0 (0)	20 (11.3)
Absent	100 (100)	157 (88.7)
X- ray results available, N (%)	100 (100)	157 (88.7)
Mild/moderate	-	82 (52.2)
Extensive	-	75 (47.8)
Cavity on chest x-ray, N (%)		157 (88.7)
Present	-	32 (20.4)
Absent	-	125 (79.6)

N - Number of subjects.

% - Percentage.

IQR - Inter quartile range.

HA - Healthy adults.

### Levels of IFN-γ and IP-10

**Figure 1:**
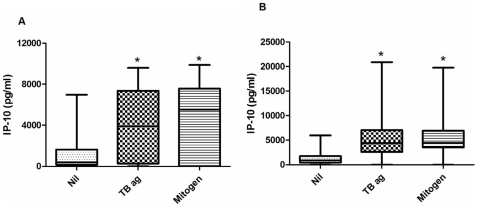
The levels of IP-10 in unstimulated, TB antigens stimulated and mitogen stimulated samples of healthy controls (a) and PTB patients (b). The levels of IP-10 in TB antigen stimulated and mitogen stimulated samples were significantly higher than unstimulated samples. Box and Whisker plots show range, inter-quartile range and median. Nil – unstimulated; TB ag – TB antigens stimulated; mitogen- mitogen stimulated samples. *significant difference p<0.05 by Kruskal- Wallis test.

**Figure 2:**
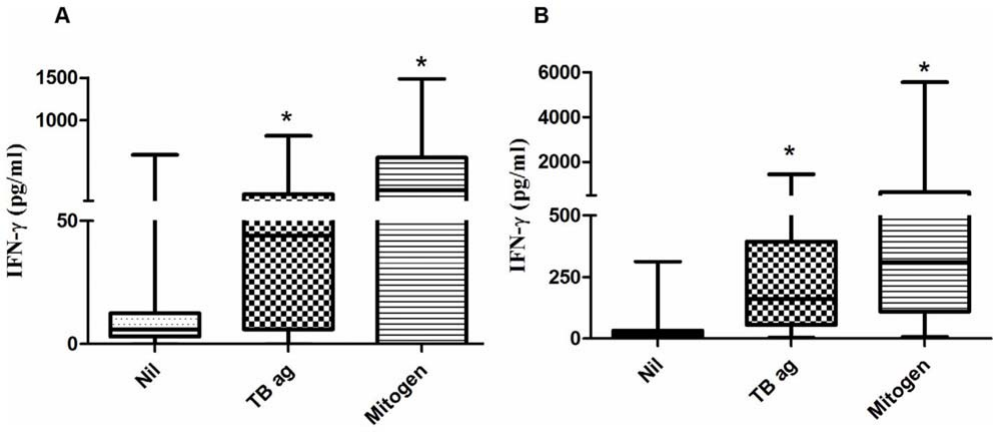
The levels of IFN-γ in unstimulated, TB antigens stimulated and mitogen stimulated samples of healthy controls (a) and PTB patients (b). The levels of **IFN-γ** in TB antigen stimulated and mitogen stimulated samples were significantly higher that unstimulated samples. Box and Whisker plots show range, inter-quartile range and median. Nil – unstimulated; TB ag – TB antigens stimulated; mitogen- mitogen stimulated samples. *significant difference p<0.05 by Kruskal- Wallis test.

The levels of IP-10 in unstimulated plasma ranged 0–6975 pg/ml (median 390 pg/ml) in healthy controls and 0–5973 pg/ml (median 898 pg/ml) in PTB patients respectively. TB specific antigens and mitogen stimulated significantly higher level of IP-10 secretion in healthy controls as well as TB patients (**Figure 1**). Levels of IFN-γ were also significantly higher in TB antigens as well as mitogen stimulated samples than unstimulated samples of healthy controls and PTB subjects (**Figure 2**).

### Cut-Off Point Determination for IP-10

**Figure 3:**
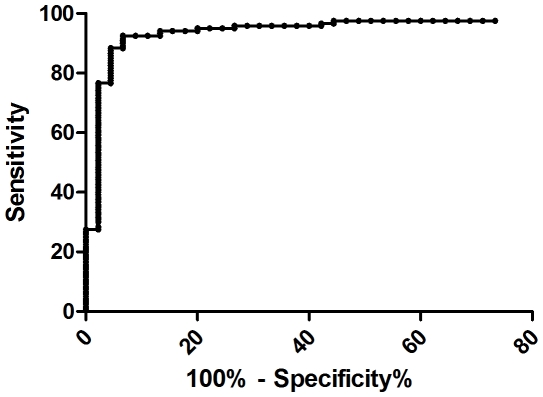
ROC curve analysis for TB antigens specific IP-10. The cut-off value for TB antigens specific IP-10 levels was determined by using QFT-IT and TST negative healthy controls as uninfected and culture confirmed TB patients as diseases subjects. The area under curve was 0.946 (95% CI: 0.905–0.987). TB- tuberculosis; IP-10-Interferon gamma inducible protein-10; QFT-IT – QuantiFERON-TB Gold in-tube; TST – Tuberculin Skin test; CI-Confidence interval.

The antigen dependent IP-10 secretion was measured by subtracting the level of IP-10 in nil tube from the TB antigen coated tube. To determine the diagnostic performance of antigen dependent IP-10 secretion, receiver-operator characteristic (ROC) curve analysis was performed. QFT-IT and TST negative healthy controls were considered as controls and culture positive PTB patients were considered as diseased. It yielded 300 pg/ml as optimum cut-off point for the antigen dependent IP-10 secretion (p<0.0001). At this cut-off point, IP-10 showed 93.5% of specificity with a sensitivity of 91.8%. (**Figure 3**). The area under curve was 0.946 (95% CI: 0.905–0.987). The 3 IP-10 positive subjects had IP-10 secretion of 520 pg/ml and 1200 pg/ml and 5120 pg/ml in response to TB specific antigens. The cut-off point for mitogen was chosen as 200 pg/ml based on the earlier observation [11].

Hence, the IP-10 results were defined as follows; the subjects with antigen dependent IP-10≥300 pg/ml (TB antigen–nil), irrespective of mitogen response were considered as positive; <300 pg/ml for antigen dependent IP-10 and ≥200 pg/ml for mitogen dependent IP-10 were considered as negative; others (<300 pg/ml of antigen dependent IP-10 for TB antigen and <200 pg/ml of mitogen dependent IP-10) were considered as indeterminate.

### Sensitivity of QFT-IT, IP-10 and TST

Of 175 PTB tested, QFT-IT and IP-10 showed the sensitivities of 88.6% (95%CI: 83.9–93.3%) and 91.4% (95%CI: 87.2–95.6%) respectively. The indeterminate results were 2.3% (95%CI: 0–4.5%) for QFT-IT and 1.2% (95%CI: 0–2.8%) for IP-10. When the indeterminate results were excluded for sensitivity calculation, QFT-IT and IP-10 yielded the diagnostic sensitivities of 90.6% (95%CI: 86.3%–94.9%) and 92.5% (95%CI: 88.6%–96.4%) respectively (Table 2). Of 119 results available, TST was positive in 88 and 82 subjects at 5 mm and 10 mm cut-off points and hence showed a sensitivity of 73.9% (95%CI: 66.0%–81.8%) and 68.9% (95%CI: 60.6%–77.2%) respectively. The sensitivity of TST was lower than QFT-IT (p<0.001) and IP-10 (p<0.001) at both 5 mm and 10 mm cut-off points. The positivity of IP-10 was 2.8% higher than QFT-IT; however, the difference between QFT-IT and IP-10 did not reach statistical significance (p = 0.383).

**Table 2:** Positivity of QFT-IT, IP-10 and TST in active TB patients.

Category	Test	N	Pos	Neg	Indt	% Sens
Total number of subjects	QFT-IT	175	155	16	4	90.6
	IP-10	175	160	13	2	92.5
	TST	119	82	37	NA	68.9
Culture positives	QFT-IT	160	141	15	4	90.4
	IP-10	160	145	13	2	91.8
	TST	110	75	35	NA	68.2
Culture negative	QFT-IT	15	14	1	0	93.3
	IP-10	15	15	0	0	100
	TST	9	7	2	NA	77.8
Smear positive	QFT-IT	149	133	12	4	91.7
	IP-10	149	136	11	2	92.5
	TST	104	73	31	NA	70.2
Smear negative	QFT-IT	26	22	4	0	84.6
	IP-10	26	24	2	0	92.3
	TST	15	9	6	NA	60.0

10 mm cut-off point was used for TST.

N - Number of subjects.

QFT-IT - QuantiFERON-TB Gold.

IP-10 - Interferon gamma inducible protein-10.

TST - Tuberculin skin test.

Pos - Positive.

Neg - Negative.

Indt - Indeterminate.

% Sens - Percentage of sensitivity, calculated after excluding indeterminate results.

The sensitivities of all three tests were similar in culture positive and negative cases **(** **Table 2** **)**. A total of 26 subjects were negative for smear microscopy and among them QFT-IT and IP-10 showed sensitivities of 84.6% and 92.3% respectively. TST results were available for 15 subjects and its sensitivity was 73.3% and 60.0% at 5 mm and 10 mm cut-off points respectively.

### Specificity of QFT-IT, IP-10 and TST

Of 100 healthy controls tested, none were indeterminate for QFT-IT or IP-10. QFT-IT and IP-10 were positive in 45 and 52 healthy controls respectively (**Table 3**); hence, they yielded the specificities of 55% (95% CI: 35.2%–54.8%) and 48% (95% CI: 38.2%–57.8%). Of 94 subjects tested for TB, TST was positive in 28 and 23 subjects at 5 mm and 10 mm cut-off points; hence showed the specificities of 70.2% (95%CI: 61.0%–79.4%) and 75.5% (95% CI: 66.8%–84.2%) respectively.

**Table 3:** Positivity of QFT-IT, IP-10 and TST in healthy adult subjects.

Tests	N	Pos	Neg	Indt	% of Spec
QFT-IT	100	45	55	0	55
IP-10	100	52	48	0	48
TST	94	23	71	0	75.5

10 mm cut-off point was used for TST.

QFT-IT - QuantiFERON-TB Gold.

IP-10 - Interferon gamma inducible protein-10.

TST - Tuberculin skin test.

N - Number of subjects.

Pos - Positive.

Neg - Negative.

Indt - Indeterminate.

% Spec - Percentage of specificity, calculated after excluding indeterminate results.

Of 66 healthy controls with TST induration <5 mm (considered to be uninfected), QFT-IT and IP-10 were positive in 21 (31.8%) and 24 (36.4%) subjects; hence showed the specificities of 68.2% and 63.6% respectively. In the 28 TST positive (at 5 mm cut-off point) subjects, QFT-IT and IP-10 were positive in 24 (85.7%) and 22 (78.6%) subjects; hence showed the specificities of 14.3% and 21.4% respectively.

### Influence of Diabetes Mellitus and Extent of Pulmonary Involvement on the Sensitivity of Tests

We then analyzed the influence of the extent of pulmonary involvement and presence of diabetes mellitus on the tests' performance. Based on the radiological abnormalities, the patients were classified into two categories; i) mild or moderate (lesions found in ≤3 zones) ii) extensive (>3 zones) [30]. The sensitivities of the tests remained similar in extensive and mild/moderate cases. The presence of cavity or diabetes mellitus also did not influence the sensitivities of any of the tests based on the statistical analysis.

### Combination of Tests

We further assessed whether the combination of tests will improve their respective sensitivities. **(** **Table 4** **)**. All the three test results were available for 117 study patients. The sensitivities of QFT-IT and IP-10 did not differ significantly between the overall population (175 subjects; 89% and 91% respectively) and subjects for whom three test results were available (117 subjects; 90% and 90% respectively).

**Table 4:** Analysis of combination of tests.

Tests	% Sens (95% CI)	% Spec (95% CI)
QFT-IT+TST	96.5 (93.2–100)	48.0 (38.0–58.0)
QFT-IT+TST+smear microscopy	98.2 (95.8–100)	48.0 (38.0–58.0)
IP-10+TST	98.3 (95.8–100)	44.8 (34.9–54.7)
IP-10+TST+smear microscopy	100.0	44.8 (34.9–54.7)
QFT-IT+IP-10	96.5 (93.2–100)	49.0 (39.0–59.0)
QFT-IT+IP-10+smear microscopy	99.1 (97.4–100)	49.0 (39.0–59.0)

10 mm cut-off point was used for TST.

Specificity was calculated as number of subjects negative for both the two tests among the total number of healthy subjects, after excluding the indeterminate results.

Sensitivity was calculated as number of subjects positive for either test among PTB patients, after excluding the indeterminate results.

QFT-IT - QuantiFERON-TB Gold.

IP-10 - Interferon gamma inducible protein-10.

TST - Tuberculin skin test.

CI - Confidence interval.

% Sen - Percentage of sensitivity.

% spe - Percentage of specificity.

Among 117 subjects, 11 (9.4%) were negative and 4 (3.4%) were indeterminate for QFT-IT. All the 4 QFT-IT indeterminate subjects were negative for TST. But 7/11 QFT-IT negative subjects were positive for TST. Hence, in the combination with TST, QFT-IT showed 93.2% (95%CI: 88.6%–97.8%) sensitivity and it was further increased to 96.5% (95%CI: 93.2%–100%), when the indeterminate results (4 subjects; 2%) were excluded.

We then analyzed the combination of IP-10 and TST. In the 117 subjects, 11 (9%) were negative and 2 (2%), indeterminate for IP-10. Both the IP-10 indeterminate subjects were negative for TST and 9 of the IP-10 negatives were positive for TST. Hence, IP-10 with the combination of TST yielded a sensitivity of 96.6% (95%CI: 93.3%–100%). Exclusion of indeterminate subjects (2 subjects; 1%) further enhanced the sensitivity to 98.3% (95%CI: 96%–100%). The combination of IP-10 and QFT-IT also failed to detect 4 subjects; hence showed the sensitivity of 93.2% (95%CI: 88.6%–97.8%) and when the indeterminate results excluded, it showed 96.5% (95%CI: 93.2%–100%).

When the combination of smear microscopy+TST+IP-10 used, they could detect 100% of active TB patients. Smear microscopy + TST + QFT-IT detected 98.2% (95%CI: 95.8%–100%) and smear microscopy + QFT-IT + IP-10 detected 99.1% (95%CI: 97.4%–100%) subjects. On the other hand, the specificity of any of two test combination was <50%.

## Discussion

This study reports that both QFT-IT and IP-10 were highly sensitive in detecting active TB patients, but poorer in specificity than TST. This is the first study evaluating IP-10 in subjects from an endemic area and comparing it with QFT-IT and TST. The combination of QFT-IT or IP-10 with TST improved their sensitivity.

Our study estimated the sensitivity of QFT-IT as being higher than the reported range in the earlier studies from high endemic countries [31]. The probable reasons for obtaining lower sensitivity for IGRA in high burden countries include HIV co-infection, advanced disease, malnutrition, host immune response and variation in the *M. tuberculosis* strain [31]. In most of the studies conducted in high burden countries, HIV positive subjects were included [14], [16] or HIV status of the study patients was unknown [15]. HIV is the one of the major influencing factors of performance of QFT-IT [32]. Our earlier study also reported the reduced sensitivity of QFT-IT in detecting active TB cases among HIV positive individuals [33]. Exclusion of HIV patients in our study may be one of the reasons for obtaining higher sensitivity for QFT-IT in contrast to earlier studies. Apart from that, the effect of genetic makeup in the population and variation in the infecting *M. tuberculosis* strains may also be alternative reasons for obtaining higher sensitivity for QFT-IT in our study than the earlier reports [34]. However, further studies are needed to confirm this aspect.

The higher levels of IP-10 were shown in serum and pleural fluid from the TB patients [35]–[37]. More recently, it was shown that secretion of IP-10 was significantly induced upon stimulation with TB specific antigens and thus also suggested as alternative marker for LTBI diagnosis [11], [17]–[20]. In this study, we found that the sensitivity of IP-10 was 2.8% higher than QFT-IT and showed less indeterminate results. IP-10 is secreted in larger quantity in response to antigens and even a small quantity of IFN-γ is enough to induce the secretion of larger quantity of IP-10 [21], [22]. A recent study conducted in Denmark population also reported that IP-10 is a potential diagnostic marker [11]. Considering the lesser number of indeterminate results and higher sensitivity of IP-10, we suggest using IP-10 as an alternative biomarker for the diagnosis of *M. tuberculosis* infection particularly where QFT-IT fails; however the utility of IP-10 would be limited by its poor specificity.

The production of IFN-γ was correlated with radiological extent of pulmonary involvement in the earlier studies [38], [39]. The secretion of IFN-γ was significantly lower in patients with far advanced radiological extent of pulmonary TB, than subjects with minimal or moderate, upon stimulation with PHA or *M. tuberculosis* strain. However, our study results suggested that the sensitivities of QFT-IT and IP-10 were not affected by radiological extent of pulmonary involvement. Diabetes mellitus is another co-morbid condition for TB patients and suppresses the expression of Th1 related cytokines [40], [41]. Hence, it is expected to be an influencing factor in immune based tests; however the trend was not observed in our study results.

The poor specificities of QFT-IT, IP-10 and TST obtained in this study were expected, due to the high number of LTBI cases in our setting. When the specificities of QFT-IT and IP-10 were compared between TST negative healthy controls and TST positive healthy controls, the specificities of both tests were higher in the former group (data not shown). This reflects the higher positivities of QFT-IT and IP-10 obtained in healthy controls due to the high number of latently infected cases.

It is reported in the earlier studies that discordance between TST and QFT-IT results were often observed [12], [42]. Taking this as an advantage, earlier studies have investigated whether the sensitivity of QFT-IT or ELISPOT have been improved when combined with TST. We applied the same approach to our data and then analyzed the sensitivity and specificity of various combinations.

We could obtain almost 90% sensitivity using QFT-IT or IP-10 alone. The analysis of different combinations showed that IP-10 and TST can detect more than 98% of active TB patients. In TB endemic countries, smear microscopy is the primary diagnostic tools to diagnose active TB patients. Any test which complements smear microscopy is highly needed in the current situation in low resource settings. Hence, we assessed whether QFT-IT, IP-10 and TST improved the sensitivity of smear microscopy. If the smear microscopy was also included in the combination, it reached 100% sensitivity. Even the other combinations like QFT-IT, TST and smear microscopy or QFT-IT, IP-10 and TST also reached >98% sensitivity.

Our study has some limitations. In this study the TST results were not available for 32.8% of PTB patients. Unexpectedly, some of the study subjects did not turn up for the TST reading. But, it unlikely influenced the performance of TST. When the subjects with TST results and without were compared, there was no significant difference observed between two groups in the demographic profile, severity of TB disease and sensitivities of QFT-IT and IP-10. In this study, we included only pulmonary TB, but not extra pulmonary TB patients. However, in our earlier study [33], we evidenced that the sensitivity of QFT-IT was similar in pulmonary and extra pulmonary TB patients. In this study, we did not include HIV positive TB patients. TB is the commonest opportunistic infection among HIV infected individuals and the progression of TB infection also is rapid [43]. Since the performance immune based diagnostic tests are expected to be attenuated in HIV infected individuals, it is time to propose different tests for HIV positive and negative individuals. After the introduction of Integrated Counseling and Testing Centre (ICTC), it is becoming a policy that TB suspected individuals have to be screened for HIV infection also and vice versa [44]. In this situation, it is possible to imply different tests for HIV positive and negative population. In addition, the prevalence of HIV infection among TB patients is low (5%) in our settings in contrast to the status in sub-Saharan African countries where 80% of TB patients were co-infected with HIV [45].

Overall, we conclude that QFT-IT and IP-10 have higher sensitivity in diagnosing the active TB cases among HIV seronegative individuals. The combination of TST, QFT-IT and IP-10 showed >95% sensitivity. The higher sensitivity of combination of QFT-IT/IP-10 and TST suggests that they may be useful in active TB diagnosis as supplement marker along with existing diagnostic tools. However their role is limited by their poor specificity due to the high prevalence LTBI in our setting.
